# Report on the Establishment of an Asylum for the Insane, Made to the Legislature of Indiana, during the Session of 1841–2

**Published:** 1842-05

**Authors:** 


Art. IV.—*Report on the Establishment of an Asylum for the Insane, made to the Legislature of Indiana, during the Session of* 1841–2.

At the late session of the Legislature of Indiana, a communication from Drs. John Evans and Isaac Fisher of Fountain County, on the subject of establishing a Lunatic Asylum, was presented and referred to the Committee on Education. Mr. Ritchey, for that committee, on the 25th of January, made a very valuable and favorable report on this matter, and eloquently urged the establishment of a State Asylum for the reception and cure of lunatics. The necessities and sufferings of this class are clearly set forth, and the advantages of such an institution to the public are plainly related in that document. In Indiana, according to the late national census, there were in 1840 one hundred and thirty-eight lunatics and idiots supported at the public cost, and four hundred and twenty-four at private charge; and the labor and service of the whole of these five hundred and sixty-two persons were lost to their families and to the State, besides the expense of maintaining them.

What proportion of these, already insane, may be restored, can only be determined by personal examination, or tested by the experiment of hospital treatment. This, however, is certain, that most cases of short standing—a year or less—may be cured; and on the other hand, that most of those whose disorder is older, cannot be cured—but yet, that even the incurable can generally be improved. It is also certain, that of all that may hereafter become insane, about nine tenths may be restored to health, usefulness, and enjoyment; and their friends and the State thereby be spared the expense of maintaining them, and also gain the advantage of their labor, and counsel, if they are properly treated. Whether the public and individuals of Indiana shall be at the expense of maintaining their insane for life, in uncured lunacy, or shall maintain them for only a few months, and then enjoy the advantage of their strength and intelligence—this is and must be the pecuniary question for the people of that State. But the pecuniary view of the matter is the least important; the health, comfort, and happiness of its citizens are of far more value to society, than their bodily service. This report, after speaking of what has been done in other States for public charities, tells the people how little has been done in Indiana.

"Your Committee cannot refrain from expressing their deep and pungent regret, that, while almost every other charitable enterprise has been undertaken and pushed forward with a zeal, perseverance and energy truly commendable, this most charitable and benevolent of all the enterprises which the spirit of philanthropy, or even Christianity, has ever recommended, should be so long neglected and postponed by the good people of our beloved State. While we have been legislating for the "*relief*" of one class after another, we have been *deaf* to the shrill and piercing cries of the maniac. While we have been spending millions upon millions upon a system of internal improvements which has involved the State in interminable difficulties, not one cent has yet been appropriated towards the establishment of asylums for the deaf and dumb, the blind, or the still more unfortunate class who have been deprived of their reason."

"While Indiana has been thus shamefully squandering her resources on works, which are likely to be viewed only as monuments of her *folly*, and neglecting to build up those institutions which the cause of humanity should have erected, as monuments of far nobler character, our own suffering fellow citizens have been knocking for admission into the charitable institutions of our sister States, and knocking in vain."*

In view of the arguments offered in this report, and of the facts which late investigations have developed, relative to the prevalence of insanity every where, and of its curability, in proper institutions, we are led to wonder that the Legislature of Indiana could have hesitated to adopt the advice of their committee, and to believe, that nothing but the inevitable necessities of these times of pecuniary embarrassment, could have postponed the establishment of a Lunatic Asylum in that State. But we may hope that the convictions of the people will demand, and their finances permit the accomplishment of this desirable and humane object in another year.

For this, we regret that the Legislature printed only five hundred copies of this report. We would rather they had published ten or twenty times that number for general distribution. As it is, we urge upon every paper in that State to reprint it; to carry its facts and arguments home to every citizen of Indiana, and thus, we are confident, that every legislator will go up to the capital instructed to vote for a State Lunatic Asylum at next session. E. J.

